# An arabic translation, reliability, and validation of Patient Health Questionnaire in a Saudi sample

**DOI:** 10.1186/s12991-017-0155-1

**Published:** 2017-09-06

**Authors:** Ahmad N. AlHadi, Deemah A. AlAteeq, Eman Al-Sharif, Hamdah M. Bawazeer, Hasan Alanazi, Abdulaziz T. AlShomrani, Raafat M. Shuqdar, Reem AlOwaybil

**Affiliations:** 1 Department of Psychiatry, College of Medicine, King Saud University, Riyadh, Saudi Arabia; 2 College of Medicine, King Saud University, Riyadh, Saudi Arabia; 3 College of Medicine, Al Imam Mohammad Ibn Saud Islamic University, Riyadh, Saudi Arabia; 4 College of Medicine, Taibah University, Madinah, Saudi Arabia; 5 SABIC Psychological Health Research & Applications Chair (SPHRAC), College of Medicine, King Saud University, PO Box 242069, Riyadh, 11322 Saudi Arabia

**Keywords:** Patient Health Questionnaire, PHQ, Arabic, Saudi Arabia, Validation

## Abstract

**Background:**

Psychological disorders including depression and anxiety are not rare in primary care clinics. The Patient Health Questionnaire (PHQ) is a clinical diagnostic tool that is widely utilized by primary health care physicians worldwide because it provides a practical in-clinic tool to screen for psychological disorders. This study evaluated the validity of the Arabic version of the PHQ in all six modules including depression, anxiety, somatic, panic, eating, and alcohol abuse disorders.

**Methods:**

This is a quantitative observational cross-sectional study that was conducted by administrating the translated Arabic version of PHQ to a sample of King Saud University students in Riyadh, Saudi Arabia.

**Results:**

The sample was 731 university students who participated in this study including 376 (51.6%) females and 354 (48.4%) males with a mean age of 21.30 years. Eight mental health experts carried out the face validation process of the PHQ Arabic version. The internal consistency reliability was measured using Cronbach’s alpha for the PHQ9, GAD7, PHQ15, and panic disorder modules. The results were 0.857, 0.763, 0.826, and 0.696, respectively. In comparison, the eating disorders and alcohol abuse modules demonstrated poor internal consistency due to small number of participants in these modules.

**Conclusion:**

This study demonstrates that the Arabic version of the PHQ is a valid and reliable tool to screen for depression, anxiety, somatic, and panic disorders in a Saudi sample.

## Background

Psychological disorders are relatively common in the community and in primary care patients. A recent study of three large primary care clinics in Riyadh, Saudi Arabia reported that nearly half of the patients expressed depressive symptoms [1]. Also, Al-Khathami and Ogbeide found that nearly one-third of the primary health clinic patients at Alkharj, Saudi Arabia demonstrated a high prevalence of psychological disorders [2]. Becker studied a primary care clinic in Riyadh, Saudi Arabia and showed that primary care physicians had poor diagnostic skills and could not accurately detect depression or somatization [3].

Many reliable self-report screening psychological instruments are available to help physicians in detecting psychological symptoms and improving their abilities to diagnose mental illness. Self-report questionnaires can provide an accurate diagnosis that is equally valid when compared to the structured interviews [4]. Thus, there is a widespread need to provide clinical instruments that can improve the diagnosis of psychological disorders at primary care clinics in Saudi Arabia.

The Patient Health Questionnaire (PHQ) is one of the most widely used clinical diagnostic instruments in primary care settings. It is valid and reliable in detecting psychological disorders. It is a self-administrated instrument that was developed in 1999 as an improved version of the original Primary Care Evaluation of Mental Disorders (PRIME-MD). It has good utility and acceptable validity compared to the original questionnaire. It measures six disorders: depression, generalized anxiety, panic, somatization, eating, and alcohol abuse disorders [4]. In the last two decades, studies have shown that the PHQ is efficient, reliable, and highly acceptable for diagnosing depression, anxiety, and somatic disorders. Löwe et al. compared the PHQ with The Hospital Anxiety and Depression Scale (HADS) and the WHO Well-being (WBI-5), tools designed for screening depression and anxiety. The PHQ had better diagnostic accuracy compared to the two well-established instruments [5]. This study used the international classification of diseases ICD-10 diagnosis criteria for depression. Conversely, the original PHQ study used the Diagnostic and Statistical Manual of Mental Disorders revised third edition and the fourth edition (DSM-III-R and DSM-IV) as criteria [4]. There is no big difference in diagnostic criteria for depression and anxiety disorders in ICD-10 and DSM-IV. Thus, it appears that the PHQ demonstrates reliable diagnostic accuracy of mental disorders whether the diagnostic criteria were based on the ICD-10 or the DSM-IV.

The PHQ has been adapted into many languages as a valuable diagnostic instrument because it exhibits high cultural sensitivity among different cultures and ethnic groups. For instance, the PHQ-9—the depression module—is a good instrument to evaluate depression cases among diverse ethnic groups in the United States such as African Americans, Chinese Americans, and Latinos [6]. Accordingly, many international adaptations of the instrument have shown that it is valid and reliable instrument. Karekla et al. examined the validity of the PHQ for a Greek population and concluded that it demonstrated good validity and reliability. Other studies also supported these findings [7]. Liu et al. [8] reported that the Chinese version of the (PHQ-9) and its subscales are valid and can accurately detect major depression in Taiwan. Furthermore, a study showed that the Patient Health Questionnaire—Somatic, Anxiety, and Depressive symptoms (PHQ-SAD), is valid and reliable for a Turkish population [9]. Many other countries and cultures adapted PHQ9 as a diagnostic and screening tool including Nepal, Nigeria, Greece, Sri Lanka, Thailand, and China [7, 10–14]. The PHQ-9 is also adapted in many medical diagnoses like AIDS, coronary artery diseases, migraine, morbid obesity, and stroke [15–19].

The PHQ was first adapted into Arabic in 2002 by a study that examined its validity in terms of detecting depression, anxiety, and somatization in primary care. The study used the Structured Clinical Interview (SCID-R) as the standard criterion and reported that the PHQ is valid in a Saudi population for diagnosing depression and somatization but not anxiety [3, 20]. However, the study did not illustrate the causes of the low sensitivity of the PHQ anxiety module. Nevertheless, its sensitivity could be improved by lowering the threshold to “several days” instead of “more than half of the days” [20]. To the best of our knowledge, no other study has shown the validity of the PHQ and its anxiety module for a Saudi population.

Other studies used the PHQ-9 to examine depression prevalence in primary care clinics. Abdelwahid and Al-shahrani reported that the prevalence of depression was 12% among patients of Family Medicine in Southeastern Saudi Arabia by using the (PHQ-9) as a detecting instrument [21]. A cross-sectional study surveyed four primary care clinics in Alkhobar, Saudi Arabia and reported that the prevalence of depression among the primary care patients was 16% by using the PHQ-9 [22]. Finally, Al-Qadhi et al. investigated the prevalence of depression in primary care clinics and reported high prevalence. They also compared the PHQ-9 and the ultra-brief version PHQ-2 and stated that the two versions are equally valid for screening depression [1]. It was evident in various studies that university students had prevalent and persistent mental health problems. In all these studies, the Arabic (Tunisian version) PHQ9 translation was used. There are no other Arabic translations available in the PHQ website other than PHQ9 and GAD7.

Until recently there was no study examining the validity of the entire PHQ with all modules in an Arabic speakers population. This study examines the validity of the PHQ in screening for depression, anxiety, somatic, panic, eating, and alcohol abuse disorders in a population of university students.

## Methods

This is a quantitative observational cross-sectional study.

### Subjects and procedure

We recruited university students from King Saud University in Riyadh, Saudi Arabia. The sample was collected through convenience sampling. We included students agreeing to participate in the study and able to read and understand Arabic. Almost all participants are Saudi and all of them are fluent in Arabic and English languages. We offered questionnaire to all university students in the medical college through representatives in each class. Data were collected from January to May 2015.

### Sample size calculation

$$n = \left( {Z_{{{\text{a} / 2}}} } \right)^{2} {{s^{2} } / {d^{2} }}$$

*s* standard deviation = 4.91 of PHQ-9 from previous study [1]; *d* the accuracy of estimate, we chose 1 difference score in the total score of PHQ-9 score; *Z*_a/2_ a normal deviate reflects the type I error which = 1.96 for 95% confidence level; Sample size = (1.96)^2^ * (4.91)^2^/(1)^2^ = 3.84 * 24.11/1 = 92.58–93 participants; So the required sample for power analysis is 93 participants.

### Measures

We used a paper and pen questionnaire. The questionnaire included demographic data and formal (not dialect) Arabic translation of the whole PHQ. PHQ consists of six modules. Depression (PHQ9-9 items), generalized anxiety (GAD7-7 items), and somatization (PHQ15-15 items) modules have items with Likert scales. Panic (15 items), eating (8 items), and alcohol abuse (5 items) modules are all Yes/No answers. The Arabic version is exactly the same structure of the original English scale. We followed the guidelines of Sousa et al. in translation, adaptation, and validation of PHQ [23]: Step 1: forward translation—translation of the PHQ into the Arabic language by two independent translators. Step 2: synthesis I—comparison of the two translated versions of the PHQ and the development of an initial translated version. Step 3: blind back-translation of the preliminary initial translated version of the PHQ from Arabic to English. Step 4: synthesis II—comparison of the two back-translated versions of the PHQ. Step 5: pilot testing of the pre-final version of the instrument in Arabic. We also did face validity by sending the pre-final version to eight referees from mental health experts.

### Ethical

The PHQ measures are in the public domain. No permission was required to reproduce, translate, display, or distribute. IRB approval from King Saud University was granted before data collection was begun.

### Data analysis

Data were analyzed using the Statistical Package for Social Sciences (SPSS) [24] (Armonk, NY, USA) version 21.0. Descriptive statistical data are presented by mean values, standard deviations, and percentages for the sociodemographic variables. Pearson’s correlation was used to assess the relationship between different variables. We used Cronbach’s alpha coefficients, corrected item-total correlation and inter-item correlation matrix analysis to assess the internal consistency reliability. A Cronbach’s alpha of ≥0.7 and item-total correlation of >0.2 were considered statistically acceptable [25]. Only statistically significant differences at *p* < 0.05 were reported.

## Results

We recruited 731 university students out of 1400 students with response rate of 52%. Almost half of them were female 376 (51.6%) with a mean age of 21.30 (SD = 1.46) years. Almost all the participants were single. Table 1 shows the demographic characteristics of the sample.

**Table 1:** Demographic characteristics

Demographic characteristics	Study sample = *n* (%) = 731 (100%)
Gender	
Male	354 (48.4%)
Female	376 (51.6%)
Age	
Mean (SD)	21.30 (1.46)
Social status	
Married	14 (1.9%)
Single	717 (98.1%)
Medical diagnoses	65 (8.9%)
Diabetes mellitus	8 (1.1%)
Hypothyroidism	3 (0.4%)
Asthma	21 (2.9%)
Irritable bowel syndrome	4 (0.5%)
Others	28 (3.8%)
Psychiatric diagnoses	19 (2.6%)
Depression	7 (1.0%)
Generalized anxiety disorder	1 (0.1%)
Obsessive compulsive disorder	6 (0.8%)
Social anxiety disorder	1 (0.1%)
More than one	4 (0.5%)

All are self-reported

### Translation process

It took approximately 2 months to finalize the process. We hired independent certified translators who are not familiar with the questionnaire. The authors of this study were the focus group that made the synthesis. All are fluent in both Arabic and English languages—some of them are mental health experts and some of them were medical students.

### Validity analysis

Face validity was carried out by a group of eight experts in mental health (psychiatrists and psychologists) fluent in Arabic and English. The final draft of the Arabic version with the original English scale and was sent via email. They completed a form stating whether they agree with the translation of each item or not and provided comments or alternative translations. They agreed on approximately 98% of the translation with few comments and suggestions. The authors reviewed all feedback points and included them in the final version of the Arabic translation.

### Reliability and item analysis

#### PHQ-9

Table 2 shows the mean scores and standard deviation for all PHQ-9 items. The most frequently endorsed item was “Feeling tired.” Suicidal ideation was the item that was endorsed the least. Cronbach’s alpha was 0.857. All items, if deleted, would decrease the total scale of Cronbach’s alpha except item 9 (suicidal ideation). All items correlated with the total scale to a good degree (lowest *r* = 0.378). Inter-item correlations range between 0.177 and 0.648 as shown in Table 3.

**Table 2:** Item statistics for PHQ-9 (major depression disorder)

PHQ-9 items	Mean	Std. deviation	Corrected item-total correlation	Cronbach’s alpha if item deleted	Change in Cronbach’s alpha if item deleted
Little interest or pleasure in doing things	1.08	0.843	0.654	0.835	−0.022
Feeling down, depressed, or hopeless	0.98	0.853	0.698	0.831	−0.026
Trouble falling or staying asleep, or sleeping too	1.27	1.042	0.558	0.846	−0.011
Feeling tired or having little energy	1.33	0.953	0.674	0.832	−0.025
Poor appetite or overeating	0.99	1.014	0.577	0.843	−0.022
Feeling bad about yourself—or that you are a failure or have let yourself or family down	0.77	0.951	0.626	0.838	−0.009
Trouble concentrating on things, such as reading the newspaper or watching TV	0.67	0.864	0.534	0.847	−0.010
Moving or speaking so slowly that other people could have or the opposite?	0.45	0.789	0.548	0.845	−0.012
Thoughts that you would be better off dead or of hurting	0.16	0.543	0.378	0.859	0.002
Total	7.71	5.433	0.400	0.857	0.000

**Table 3:** Inter-item correlation matrix for PHQ-9 (major depression disorder)

PHQ-9 items	Item 1	Item 2	Item 3	Item 4	Item 5	Item 6	Item 7	Item 8	Item 9
Little interest or pleasure in doing things	1.000	–	–	–	–	–	–	–	–
Feeling down, depressed, or hopeless	0.648	1.000	–	–	–	–	–	–	–
Trouble falling or staying asleep, or sleeping too	0.431	0.439	1.000	–	–	–	–	–	–
Feeling tired or having little energy	0.553	0.507	0.583	1.000	–	–	–	–	–
Poor appetite or overeating	0.397	0.454	0.410	0.468	1.000	–	–	–	–
Feeling bad about yourself or that you are a failure or have let yourself or family down	0.485	0.578	0.356	0.444	0.445	1.000	–	–	–
Trouble concentrating on things, such as reading the newspaper or watching TV	0.377	0.376	0.345	0.417	0.368	0.398	1.000	–	–
Moving or speaking so slowly that other people could have or the opposite?	0.389	0.421	0.290	0.388	0.383	0.403	0.462	1.000	–
Thoughts that you would be better off dead or of hurting	0.265	0.370	0.177	0.236	0.223	0.347	0.219	0.348	1.000

#### GAD-7

Table 4 shows the mean scores and standard deviation for all GAD7 items. The most frequently endorsed item was “Feeling nervous, anxious, on edge, or worrying a lot about different things.” “Feeling restless so that it is hard to sit still” was the item that was endorsed the least. Cronbach’s alpha was 0.763. All items, if deleted, would decrease the total scale of Cronbach’s alpha. All items correlated with the total scale to a good degree (lowest *r* = 0.410). Inter-item correlations range between 0.204 and 0.426 as shown in Table 5.

**Table 4:** Item statistics GAD-7 generalized anxiety disorder

GAD-7 items	Mean	Std. deviation	Corrected item-total correlation	Cronbach’s alpha if item deleted	Change in Cronbach’s alpha if item deleted
Feeling nervous, anxious, on edge, or worrying a lot about different things	1.31	0.486	0.423	0.747	−0.016
Feeling restless so that it is hard to sit still	0.49	0.646	0.483	0.733	−0.030
Getting tired very easily	0.91	0.720	0.595	0.707	−0.056
Muscle tension, aches, or soreness	0.55	0.674	0.441	0.742	−0.021
Trouble falling asleep or staying asleep	0.92	0.743	0.410	0.751	−0.012
Trouble concentrating on things, such as reading a book or watching TV	0.71	0.714	0.529	0.723	−0.040
Becoming easily annoyed or irritable	1.06	0.662	0.505	0.729	−0.034
Total	5.95	3.004	0.317	0.763	0.000

**Table 5:** Inter-item correlation matrix GAD-7 generalized anxiety disorder

GAD-7 items	Item 1	Item 2	Item 3	Item 4	Item 5	Item 6	Item 7
Feeling nervous, anxious, on edge, or worrying a lot about different things	1.000	–	–	–	–	–	–
Feeling restless so that it is hard to sit still	0.314	1.000	–	–	–	–	–
Getting tired very easily	0.336	0.369	1.000	–	–	–	–
Muscle tension, aches, or soreness	0.204	0.288	0.426	1.000	–	–	–
Trouble falling asleep or staying asleep	0.224	0.236	0.352	0.240	1.000	–	–
Trouble concentrating on things, such as reading a book or watching TV	0.284	0.383	0.389	0.297	0.319	1.000	–
Becoming easily annoyed or irritable	0.332	0.324	0.410	0.277	0.260	0.384	1.000

#### PHQ-15

We calculated it as instructed in PHQ instruction manual [26] by assigning scores of 0, 1, and 2 to the response categories of (not at all, bothered a little, and bothered a lot) for the 13 somatic symptoms of the PHQ (items 1a-1 m). Also, 2 items from the PHQ-9 were added (sleep and energy) and scored 0 (not at all), 1 (several days) or 2 (more than half the days or nearly every day). Table 6 shows the mean scores and standard deviation for all PHQ-15 items. The most frequently endorsed item was “Feeling tired or low energy.” “Pain or problems during sexual intercourse” was the item that was endorsed the least. Cronbach’s alpha was 0.826. All items, if deleted, would decrease the total scale Cronbach’s alpha with the exception of “Pain or problems during sexual intercourse.” All items correlated with the total scale to a good degree (lowest *r* = 0.207) except item 5 “Pain or problems during sexual intercourse” with *r* = 0.032. Inter-item correlations range between −0.040 and 0.588 as shown in Table 7.

**Table 6:** Item statistics for PHQ-15 (Somatization Disorder Scale)

PHQ-15 items	Mean	Std. deviation	Corrected item-total correlation	Cronbach’s alpha if item deleted	Change in Cronbach’s alpha if item deleted
Stomach pain	0.73	0.692	0.515	0.811	−0.015
Back pain	0.75	0.731	0.454	0.815	−0.011
Pain in your arms, legs, or joints (knees, hips, etc.)	0.69	0.717	0.498	0.812	−0.014
Menstrual cramps or other problems with your	0.57	0.761	0.460	0.815	−0.011
Pain or problems during sexual intercourse	0.00	0.053	0.032	0.830	0.004
Headaches	0.91	0.707	0.417	0.818	−0.008
Chest pain	0.28	0.513	0.353	0.821	−0.005
Dizziness	0.49	0.630	0.502	0.812	−0.014
Fainting spells	0.05	0.257	0.207	0.827	0.001
Feeling your heart pound or race	0.60	0.684	0.525	0.811	−0.015
Shortness of breath	0.40	0.600	0.499	0.813	−0.013
Constipation, loose bowels, or diarrhea	0.73	0.737	0.514	0.811	−0.015
Nausea, gas, or indigestion	0.80	0.767	0.580	0.806	−0.020
Trouble falling or staying asleep, or sleeping too	1.11	0.819	0.398	0.821	−0.005
Feeling tired or having little energy	1.17	0.734	0.509	0.811	−0.015
Total	9.28	5.331	0.224	0.826	0.000

**Table 7:** Inter-item correlation matrix for PHQ-15 (Somatization Disorder Scale)

PHQ-15 items	Item 1	Item 2	Item 3	Item 4	Item 5	Item 6	Item 7	Item 8	Item 9	Item 10	Item 11	Item 12	Item 13	Item 14	Item 15
Stomach pain	1.000	–	–	–	–	–	–	–	–	–	–	–	–	–	–
Back pain	0.346	1.000	–	–	–	–	–	–	–	–	–	–	–	–	–
Pain in your arms, legs, or joints (knees, hips, etc.)	0.288	0.458	1.000	–	–	–	–	–	–	–	–	–	–	–	–
Menstrual cramps or other problems with your periods	0.328	0.255	0.277	1.000	–	–	–	–	–	–	–	–	–	–	–
Pain or problems during sexual intercourse	0.021	0.019	0.060	0.101	1.000	–	–	–	–	–	–	–	–	–	–
Headaches	0.222	0.199	0.253	0.289	−0.031	1.000	–	–	–	–	–	–	–	–	–
Chest pain	0.198	0.221	0.223	0.117	−0.029	0.181	1.000	–	–	–	–	–	–	–	–
Dizziness	0.245	0.307	0.321	0.273	0.001	0.323	0.214	1.000	–	–	–	–	–	–	–
Fainting spells	0.099	0.135	0.130	0.098	−0.011	0.051	0.191	0.172	1.000	–	–	–	–	–	–
Feeling your heart pound or race	0.272	0.222	0.293	0.249	−0.008	0.294	0.289	0.369	0.183	1.000	–	–	–	–	–
Shortness of breath	0.241	0.262	0.273	0.230	0.054	0.255	0.346	0.354	0.205	0.484	1.000	–	–	–	–
Constipation, loose bowels, or diarrhea	0.424	0.221	0.262	0.310	0.020	0.219	0.199	0.234	0.154	0.314	0.260	1.000	–	–	–
Nausea, gas, or indigestion	0.505	0.264	0.351	0.351	0.049	0.234	0.221	0.300	0.120	0.327	0.315	0.588	1.000	–	–
Trouble falling or staying asleep, or sleeping too	0.226	0.182	0.206	0.198	−0.040	0.236	0.141	0.224	−0.009	0.256	0.201	0.203	0.229	1.000	–
Feeling tired or having little energy	0.248	0.248	0.257	0.304	0.024	0.257	0.155	0.325	0.094	0.308	0.277	0.289	0.295	0.525	1.000

### Other scales

Panic disorder, eating disorders, and alcohol abuse sections were all Yes/No answers. It is different than PHQ-9, GAD-7, and PHQ-15, which are Likert scale answers.

The panic disorder scale consists of 15 items. It started with a single question “In the last 4 weeks, have you had an anxiety attack suddenly feeling fear or panic?” If the subject answered “No” then there was no need to complete the rest of the items. The next 3 items asked about attack details, and the next 11 items focused on the physical symptoms during attacks. It measures the diagnosis not the severity. Cronbach’s alpha was 0.696. All items, if deleted, would decrease the total scale of Cronbach’s alpha except “Have you had an anxiety attack—suddenly fear or panic?”, “Has this ever happened before?” and “Did you tremble or shake?” they will increase alpha to 0.700, 0.709, and 0.707, respectively.

Bulimia nervosa and binge eating disorder have 8 items. Cronbach’s alpha was 0.110. Again, it measures the diagnosis not the severity. This scale started with 2 items. If any were answered with a “Yes” then the participant needs to proceed; otherwise, he stops.

The alcohol abuse scale consisted of 5 items. Cronbach’s alpha was 0.280. This scale starts with a question: “Do you ever drink alcohol (including beer or wine)?” If participant checks “NO” then he needs to stop and not answer the remaining 5 items.

## Discussion

This study evaluated the validity and reliability of the PHQ in a sample of university students. The PHQ is very helpful tool for diagnosis and also for severity measures for many psychiatric disorders. Some studies already used the PHQ-9, GAD-7, and PHQ-15 in Saudi Arabia. Most of them used a straightforward translation method or the Tunisian Arabic version of the PHQ-9 in PHQ screeners website [22].

The PHQ-9 showed good internal consistency with Cronbach’s alpha of 0.857. Usually, self-reported scales are considered to have good reliability if Cronbach’s alpha ranges between 0.70 and 0.95 [25]. This is consistent with the results of PHQ in the US where the alpha coefficient ranged from 0.79 to 0.89 [6, 27]. It also agrees with a Nigerian study that showed the alpha PHQ9 to be 0.85 [11]. The suicidal ideation item is the only item that if deleted will increase the reliability by 0.002. This small increment does not motivate removing the item. However, there is another version of PHQ-9 without this item. It is called PHQ-8 and is used mainly in non-depression research studies [26]. All other items correlated to the total scale nicely. Ideally, the average inter-item correlation for a set of items is better between 0.20 and 0.40 [28].

The GAD-7 reliability is acceptable with Cronbach’s alpha of 0.763, and all the items are nicely correlated with the total scale and also to each other. The PHQ-SADS includes PHQ-9, GAD-7, and PHQ-15 measures plus panic measure from the original PHQ. However, GAD7 in PHQ is not the same as in PHQ-SADS in many points. First, only 3 items are the same in both versions of GAD7. Second, answers in GAD7 of the PHQ include 3 options “Not at all, several days, and more than half the days” where “nearly every day” is added in GAD-7 of PHQ-SADS. Third, duration of symptoms is 4 weeks in GAD-7 of PHQ, while it is 2 weeks in GAD-7 of PHQ-SADS. We used GAD7 of the PHQ and found it to be reliable. We do not know which version of GAD7 was used in Becker et al. which found that the anxiety scale is not valid in Arabic (Saudi Arabia) [20]. We tried to get this information or to get the Arabic version that was used but we were unable because of no response from the authors.

The PHQ-15 somatization scale showed good internal consistency with Cronbach’s alpha with 0.826. A Greek study found PHQ-15 Cronbach’s alpha to be 0.73 for women and 0.71 for men [29]. In a Swedish sample, the alpha coefficients of the PHQ-15 ranged from 0.75 to 0.85 between study groups [30]. Becker et al. found sensitivity and specificity for Arabic somatization scale 0.65 and 0.96, respectively [20]. All PHQ-15 items are nicely correlated with the total scale with the exception of item 5 “Pain or problems during sexual intercourse.” This item showed poor correlation with other items in inter-item correlations. The inter-item correlations of the PHQ-15 showed 5 negative scores out of 15 scores—none of these were >0.2. This could be because almost all participants were single (98.1%). Only 10 of the participants answered this question, and the rest of participants left it blank. Eight chose “not at all,” and only two chose “bothered a little.” To correctly test this item, it is better to select a cohort with more married participants.

### Other scales

Panic disorder scale showed acceptable internal consistency with alpha = 0.696. This confirms a Greek study that found alpha = 0.73 [7]. We believe that the items—that if they are deleted may increase the reliability—are worth to stay in the scale. First, the increase is not that much (a 0.013 maximum). Second, they are crucial—especially the first one, which is the gate for the scale. Also, the second item focuses on attack recurrence, which is a criterion for panic disorder diagnosis. The least important item is shaking and tremor although it is among common physical symptoms in the panic attack.

Unfortunately, eating disorders and alcohol abuse modules have low internal consistency scores. This can be explained by the small number who answered these modules and small number of items. The Cronbach alpha depends on the sample size and on the items number in the scale. Small item numbers (<10) have Cronbach alpha values that are usually very small [28]. Eating disorder modules have only 8 items and were answered by only 41 participants (5.6%). This is not consistent with other studies. For example, the Greek study found that Cronbach’s alpha for an eating disorder module is 0.70 [7].

The alcohol abuse scale showed low reliability. Alcohol drinking is prohibited and illegal in Saudi Arabia. This low reliability score could be due to the low participation rate in this section. It has only 5 items, and only 6 participants (<1%) answered the scale. All were male. Although no females answered the scale, the last item in the scale stated “Driving under the influence,” and this cannot be measured in Saudi women because it is illegal for females to drive in Saudi Arabia.

## Conclusion

The PHQ Scale is a widely used tool with many translations worldwide. The Arabic version of the PHQ is a valid and reliable measure to screen depression, anxiety, somatic, and panic disorders in a Saudi sample. Eating disorders and alcohol abuse modules need to be administered on different samples to have more participation. We hope our study will encourage researchers and practitioners to conduct more studies in Saudi Arabia regarding mental health disorders.

## Limitations

Our study has several limitations. First, the study was conducted among university students; therefore, it cannot be generalized. Second, it would be better if the reliability was examined through test–retest and not limited to the internal consistency tests. Third, we did not do convergent validity by comparing the scale to another gold standard. Fourth, there were few participants in the eating disorder and alcohol abuse modules.

## Clinical implications

The application of validated and reliable Arabic PHQ will have a better impact in the recognition and detection of various mental health disorders that are under-diagnosed.

## Abbreviations

ANOVA: analysis of variance
DSM: Diagnostic and Statistical Manual of Mental Disorders
GAD7: PHQ—generalized anxiety disorder items
HADS: Hospital Anxiety and Depression Scale
ICD: international classification of diseases
PHQ-SAD: Patient Health Questionnaire—Somatic, Anxiety, and Depressive symptoms
PHQ: Patient Health Questionnaire
PHQ15: PHQ—somatization disorder items
PHQ9: PHQ—major depressive disorder items
PRIME-MD: Primary Care Evaluation of Mental Disorders
SCID-R: Structured Clinical Interview
WBI-5: WHO Well-being Inventory
